# The importance of recognizing paraneoplastic symptoms: a case report of Neuroendocrine Small Cell Carcinoma of the Endometrium presenting as Paraneoplastic Cushing’s Syndrome.

**Published:** 2017-06

**Authors:** A Doukhopelnikoff, G Debrock, T Steelandt, ETM De Jonge

**Affiliations:** KULeuven, Faculty of Medicine, Gasthuisberg, Herestraat 49, 3000 Leuven, Belgium; Department of Oncology, Ziekenhuis Oost-Limburg Campus St Jan, Schiepse Bos 6, 3600 Genk, Belgium; Department of Pathology, Ziekenhuis Oost-Limburg Campus St Jan, Schiepse Bos 6, 3600 Genk, Belgium; Department of Obstetrics & Gynaecology, Ziekenhuis Oost-Limburg Campus St Jan, Schiepse Bos 6, 3600 Genk, Belgium.

**Keywords:** Corpus uteri, Carcinoma, Cushing’s syndrome, Paraneoplastic syndrome, Small cell carcinoma, euroendocrine

## Abstract

This is the second well documented case of paraneoplastic Cushing’s syndrome arising from a small cell carcinoma of the endometrium described in English literature. This tumour has an aggressive biological behaviour and early detection provides the only opportunity for long-term survival. In that regard recognition of associated paraneo- plastic features might be helpful.

## Introduction

Small cell carcinoma (SCC) is mostly associated with lung cancer as small cell lung carcinoma (SCLC) and accounts for about 13% of all lung cancer (Govindan et al., 2006). Extrapulmonary small cell carcinoma (EPSCCA), SCC that arises from an organ other than the lungs, is rare and can emerge from virtually any organ. The most common origins are: gastrointestinal (33%) followed by genitourinary (20%), head and neck (11%), and breast (10%) (Howard S et al., 2011). EPSCCA like SCLC is of very aggressive nature and often with morbid prognosis. In the genital tract the cervix is the most common site of origin followed by the ovaries. There only exist few case reports about endometrial SCC. (van der Heijden et al., 2005). A variable portion of cervical SCC’s exhibit neuro-endocrine differentiation (Gersell et al., 1988). Paraneoplastic syndromes due to ectopic production of hormones such as SIADH (syndrome of inappropriate antidiuretic hormone secretion) and Cushing’s syndrome (ectopic adrenocorticotropic hormone (ACTH) production) are thus not uncommon in cervical SCC. We report the case of an endometrial SCC with paraneoplastic Cushing’s syndrome which has only been described once before in English literature (Sato et al., 2009).

## Case report

A 32-year-old female, mother of two children, presented with persistent migraine-like headache, palpitations, progressive nausea and vomiting, photo- and sonophobia, menometrorrhagia and concomitant general fatigue. Clinical assessment changed markedly over the course of the following weeks whilst under exploration: she steadily became severely hypertensive, developed a distended abdomen and peripheral oedema, she gained nineteen kilograms in bodyweight. In addition acne, hyperpigmentation, and facial hair growth developed insidiously.

Further exploration revealed a severe hyponatremia (SIADH secondary to the use of carbamazepin for migraine) causing the headaches, photo- and sonophobia, nausea and vomiting as well as a severe hypokalemia explaining the palpitations and premature supraventricular complexes, ST-segment and T- wave abnormalities on ECG. As she started to show more pronounced systemic signs (hyperpigmentation, oedema, acne) laboratory tests revealed elevated cortisolemia levels (63.4 μg/dl; normal range: 6.2 - 18 μg/dl), abnormal 24 hours cortisoluria (7531 μg/24 hours; normal range: 3.5 - 45 μg/24 hours), increased ACTH level (846.1 ng/L; normal range: 10 - 60 ng/L) and an abnormal dexamethasone suppression test overnight. The diagnosis of Cushing’s syndrome was made. Urinary potassium levels of 19.39 mmol/L and a 24-hour kaliuresis of 80 mmol (normal range, 25 - 125 mmol/24h) were found, even while hypokalemia was severe. The transtubular potassium gradient was 12.7 (normal range <3), which indicated a mineralocorticoid effect. Following a negative MRI of the pituitary, the elevated plasma ACTH levels were indicative of ectopic production.

**Figure 1:**
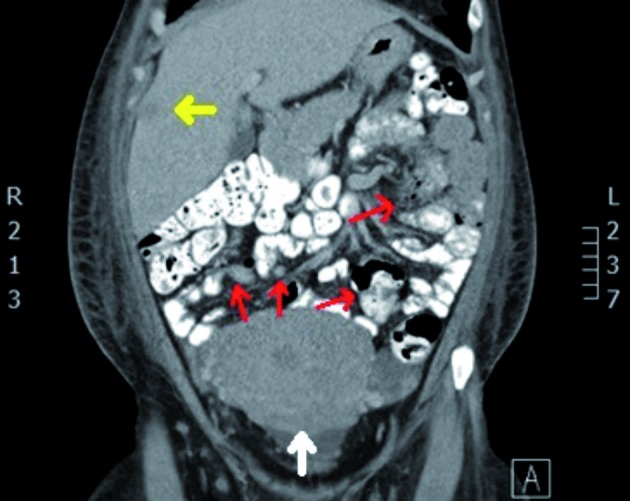
— Abdominal CT scan: frontal section. Voluminous uterus (white arrow), liver metastasis (between lobe 5 and 8, yellow arrow) and mesenteric metastasis (red arrows).

Initial therapy consisted of ketoconazole as suppression of cortisol production, together with one (empirical) gift of somatostatin, perindopril as antihypertensive drug, and low molecular weight heparins in preventive dosage for the elevated risk of thrombosis. In search of the ectopic ACTH production locus, CT scan revealed a voluminous pelvic tumour widely disseminated to liver and peritoneum with multiple retroperitoneal adenopathies (Fig.1). Multiple sub-centimetrical pleural noduli were seen in the posterior upper right lung lobe.

**Figure 2:**
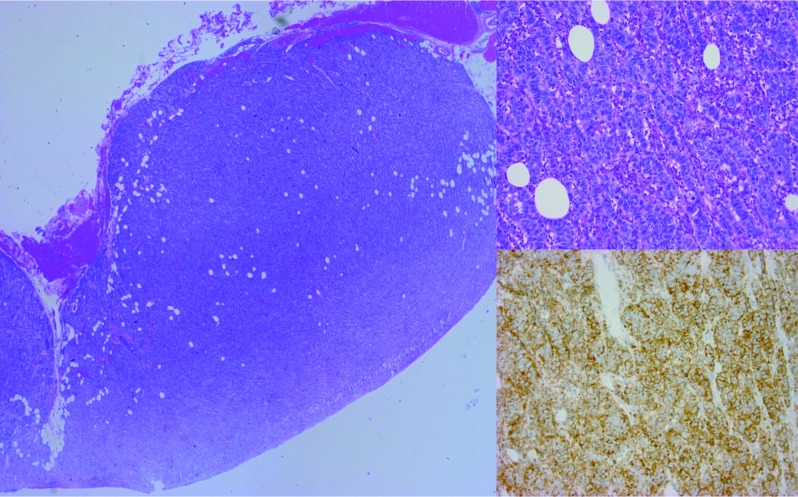
— Left: Low power magnification of a peritoneal tumorectomy. Upper right: 200x Magnification of the nodule showing a trabecular growth pattern of medium-sized cells with scant cytoplasm. Lower right: Cell stain positive for CD56/NCAM.

A gynaecologic oncologist was consulted. Pelvic examination showed a hypertrophic cervix with pronounced smooth nodular aspect and multiple vaginal lesions with the same nodular appearance. The uterus was enlarged with multiple nodules presenting clinically as a 16 weeks pregnancy sized myomatous uterus with free parametria. An explorative diagnostic laparoscopy was performed. Internal inspection of the pelvis confirmed the presence of an enlarged, pale and nodular uterus whitish surrounded by multiple (>50) peritoneal implants with similar appearance. Implants were also noted in the omentum and at the right diaphragm with moderate ascites. The ovaries were normal. Biopsies of the cervix and two peritoneal implants were taken, revealing a high-grade neuroendocrine carcinoma in both the peritoneal implants and cervix (though subepithelial location in the cervix). This conclusion was based on histomorphological features (trabecular proliferation of monotonous population of small cells with hyperchromatic nuclei and moulding; high mitotic and apoptotic activity) combined with positive neuroendocrine markers such as CD56/NCAM (neural cell adhesion molecule) (Fig. 2), synaptophysine, chromogranine (with positive external control) and positive cytoplasmic dot-like pankeratin staining. The final diagnosis of FIGO stage IVb endometrial carcinoma was made.

Serum tumour markers neuron-specific enolase (NSE) and chromogranin were positive respectively 285 ng/ml (normal range <15 ng/ mL) and 544 μg/L (normal range <36.4 μg/L). Palliative chemotherapy with a combination of cisplatin and etoposide was proposed. Prior to the first administration of chemotherapy the patient developed erysipelas of the right leg. Intravenous anti- biotics were administered and chemotherapy was started. The next day her condition worsened with persistent fever and a rising CRP. She developed a septic shock and in spite of antibiotherapy and maximal supportive therapy she died 48 hours after the initiation of chemotherapy.

## Review of the literature and discussion

In the light of the importance of early detection of (gynaecologic) neoplasms, research about reported paraneoplastic syndromes associated with cancer of the endometrium was conducted in the literature with (combinations of) keywords: “endometrial”, “endometrium”, “carcinoma”, “paraneoplastic”, “syndrome”. Search databases PubMed, Google scholar and trip database were used. Our findings (in order of most literature found first): retinopathy (Sekiguchi et al., 1998), hypercalcemia (Hiller et al., 1989), vasculitic neuropathy (Oh et al., 1991), membranous glomerulonephritis (Meydanli et al., 2003), cerebellar degeneration (Brock et al., 2001), multicentric reticulohistiocytosis (Malik et al., 2005), acanthosis nigricans (of the palms) (Mekhail and Markman, 2002), palmar fasciitis and arthritis (Docquier et al., 2002), multiple hamarthoma syndrome (MHS) with or without sign of Leser-Trélat (Aylesworth and Vance, 1982), uveal melanocytic proliferation (Chahud et al., 2001), pemphigus (Kennedy and Dawe, 2009), digital ischemia (Mahler et al., 1999) and dermatomyositis (Wada et al., 2014). Knowledge of these particular syndromes is useful since early detection (syndrome sometimes present even before gynaecologic symptoms) almost always leads to better prognosis. This is even more true when occurrence is at premenopausal age where postmenopausal bleeding cannot be present as red flag, or when the tumour is of very aggressive nature (e.g. SCC). Early detection provides the only opportunity for long-term survival in patients with small cell carcinoma of the endometrium (Katahira et al., 2004). Only one report of endometrial carcinoma with paraneoplastic Cushing’s syndrome was found in the literature (Sato et al., 2009). The case report also described a FIGO stage IVb SCC of the endometrium

Neuroendocrine SCC are characterized by aggressive behaviour (Stachs et al., 2005) with extensive local invasion and early metastasis to distant organs (Crowder and Tuller, 2007). Katahira et al. (2004) described a series of 43 cases of endometrial SCC with a mean age of 60 years with only four cases at age 40 or less. Noticeable was that three out of these four cases got diagnosed as FIGO stage IVb. This feature (late presentation in premenopausal women) is potentially due to the lack of a well-recognized alarming sign such as postmenopausal bleeding or due to a more aggressive biological tumour behaviour. In contrast to women suffering from endometrial adenocarcinoma where low parity is frequent, SCC of the endometrium occurs mostly (including our case) in parous women (Katahira et al., 2004).

van Hoeven et al. (1995) proposed three diagnostic criteria for small cell neuroendocrine endometrial carcinoma, of which all three are fulfilled in our case: (1) unequivocal evidence of endometrial origin; (2) a dense sheet-like growth of morphologically similar small to intermediate-sized tumour cells examined by standard H&E-stained sections; and (3) immunohistochemical reactivity for one or more neuroendocrine markers. Many cases of SCC of the endometrium present as a mixed entity with presence of other tumour phenotypes such as adenocarcinoma though cases of ‘pure’ SCC are not rare. In 1994, Huntsman et al. published a clinicopathological study of 16 cases of endometrial SCC, describing its clinical presentation. Menorrhagia and abdominal distension were the most commonly noted signs in premenopausal women whilst vaginal involvement (2 patients), extra-uterine spread (8 patients) and widespread intra-abdominal metastasis (4 patients) were surely not infrequent. The uterus was often described as bulky (13 patients) and with pale, in two cases almost “fish-flesh”-like appearance. In four cases the cervix was grossly involved by tumour but the bulk of the tumour was present in the corpus. Nine (of 11 tested patients) were NSE positive. Six patients out of 16 were FIGO stage IVb. This allows us to conclude that our case’s features (both clinical and macropathological) accord very well to the general presentation and appearance of endometrial SCC.

Nieman et al. (2008) defined a clinical guideline for diagnosis of Cushing’s syndrome in which all possible clinical features of Cushing’s syndrome are stipulated. Cushing’s syndrome is more likely to be present when a large number of signs and symptoms are present and have accumulated over time. This is in line with the story of our patient who progressively struggled with more and more of these symptoms. Centripetal fat distribution with striae, peripheral oedema (primarily of the legs), hypertension, weight gain, hirsutism, muscle weakness, depressed mood, hyperpigmentation, fatigue, facial acne, unusual infection and hypokalemia were all present, eventually. Atrophic skin, bruising and buffalo hump were not present as these symptoms usually develop under more chronic exposure to high levels of cortisol

Hyperpigmentation is often found as a clinical sign in patients with high plasma ACTH-levels such as in Addison’s disease or Cushing’s syndrome. When neuroendocrine tumours or the pituitary gland produce excessive amounts of ACTH via the POMC (pro-opiomelanocortin) pathway, they cause hyperpigmentation of the skin because in order to produce ACTH, POMC has to be cleaved into α-MSH (melanocyte stimulating hormone), ACTH and endorphin which are all three stimulatory agents for skin pigmentation (Yamaguchi et al., 2009).

Torpy et al. (2002) showed with 58 cases of ectopic ACTH producing Cushing’s syndrome that the prevalence of hypokalemia in this population was about 57% (38 cases). This is much higher than the prevalence of hypokalemia in pituitary-dependent Cushing’s (about 10%). The cause of hypokalemia in Cushing’s syndrome is not yet fully understood. It is known, however, that hypokalemia strongly correlates with plasma cortisol level. This hypothesis is explained by the cortisol-related mineralocorticoid activity that arises in the event of high levels of cortisoluria. The detailed mechanism is as follows: cortisol acts as a mineralocorticoid, as its in vitro binding affinity to the mineralocorticoid receptor is equal to that of aldosterone. The difference is that cortisol is broken down by 11β-hydroxysteroid- dehydrogenase (11β-HSD2) in the renal tubule as protection for the mineralocorticoid receptor. However, 11β-HSD2 can become saturated when cortisol is excessively present in plasma (and renal tubule). This gives some intact cortisol the chance to exert mineralocorticoid effect (Funder et al., 1988). Mineralocorticoids such as aldosterone mediate sodium reuptake and potassium secretion through the up-regulation of serosal Na/K-ATPase and luminal sodium-ion-channels. While acute ACTH administration transiently stimulates aldosterone secretion, both aldosterone and plasma renin activities are normal to low in Cushing’s syndrome, suggesting that the aldosterone/renine system is not the cause of the mineralocorticoid type of hypertension and hypokalemia which may be seen (Torpy et al., 2002).

Our patient had a very high cortisoluria of 7531 μg / 24 hours (normal range; 3.5 - 45 μg/24 h) and a transtubular potassium gradient of 12.7 (normal range <3), despite hypokalemia as low as 1.69 mmol/L. Although patient’s oedema was treated with a combination of a thiazide- and a potassium- sparing diuretic for 3 weeks, this can never give rise to a hypokalemia this severe. As we see no other possible cause of patient’s severe hypokalemia we propose the cortisol-related mineralocorticoid activity that arises in the event of high levels of cortisoluria as full explanatory mechanism of hypokalemia in Cushing’s syndrome. Biller et al. (2008) propose two possible types of therapy for paraneoplastic Cushing syndrome with ectopic ACTH production. Namely: tumour- directed therapy and adrenal-directed therapy. For our patient, tumour-directed therapy involved somatostatin analogs (possible expression of somatostatin receptors) and systemic chemotherapy as tumour stage was already advanced. Adrenal-directed therapy is aimed to block cortisol production by means of medication or bilateral adrenalectomy. In our case, ketoconazole was started since treatment with metyrapone (the other first line drug) is known to be associated with hirsutism (a most unwelcome side effect for women), hypokalemia, hypertension and oedema (these three already strongly present). Bilateral adrenalectomy could not be performed because of high morbidity.

Nieman et al. (2008) discussed morbidity and mortality of Cushing’s syndrome and dedicate most deaths to vascular (myocardial infarction, cerebrovascular accident) or infectious complications. In our case, ECG showed signs of possible inferior myocardial infarction and erysipelas was also present in the end. Kumar (1984) described a case of a 23-year-old woman with SCC of the endometrium who developed severe leukopenia and thrombocytopenia (believed to be) secondary to invasion of her bone marrow by the neoplasm. This is unmistakably similar to our case where neutropenia and thrombocytopenia developed, merely one day after initiation of chemotherapy (hematotoxicity secondary to cytostatics classically arises after a minimum of two weeks). Bone marrow infiltration as advanced stage of tumoural spread is therefore strongly suspected in our case (Table 1). Oedema is to be seen as one of the principal risk factors in developing erysipelas (Dupuy et al., 1999). In combination with high cortisolemia, neutropenia and incidental site of entry, the oedema of the lower extremities probably led to rapidly progressive erysipelas, septic shock and eventually to the patient’s death. It can thus be said that the malignant process and paraneoplastic syndrome caused complications, which, in unison, led to the swift demise of our patient.

**Table I:** — Overview of relevant laboratory parameters over time starting at the day of presentation (day 1) until the day of demise (day 58).

Test	Normal range	Day								
		1	7	29	31	43	55	56	57	58
Natrium (mmol/L)	135-145	117^(1)^	130	138	144	139	146	144	146	140
Potassium (mmol/L)	3.5-4.5	3.80	3.68	1.69^(2)^	2.55	3.51	2.30	2.59	2.17	3.30
Cortisol (μg/dl)	2.7-10.4					63.4		63.4		
ACTH (ng/L)	10-60				166.9	214.2	425.3	846.1		
WBC (x 10^3^)	4.5-11	6.0					10.9	10.1	1.1	0.1
Platelets (x 10^3^)	150-400	179					89	72	34	9
CRP (mg/L)	<5	1.2					16.3	68.2	298.2	315.9

(1): Stop carbamazepine for suspected SIADH

(2): On ICU with ECG monitoring; start Potassium substitution and thiazide diuretics on hold

Because so-called extrapulmonary small cell carcinoma (EPSCCA) is a relatively rare disease that mimics small cell lung carcinoma in its response to treatment and survival patterns, it appeared advisable to follow similar treatment guidelines (Galanis et al., 1997). Therefore, etoposide and cisplatin chemotherapy was given to our patient as would be given to metastatic SCC of the lung.

## Conclusion

In conclusion we can say that neuroendocrine SCC of the endometrium is extremely rare and of poor prognosis considering its aggressive nature with rapid invasion and extra-uterine spread. Early diagnosis is vital: timely recognition of a paraneoplastic syndrome as an early sign is unlikely, but it can save lives. This is the second well-documented case of paraneoplastic Cushing’s syndrome arising from a small cell carcinoma of the endometrium described in English literature. It confirms the existence of this clinical entity and shows that combination of neoplasia and paraneoplastic syndrome can interfere with standard treatment and lead to unexpected complications and clinical course.
